# The Institutional Worker

**Published:** 1919-10-04

**Authors:** 


THE
### INSTITUTIONAL WORKER
**Being a Special Supplement to "The Hospital"**

### OUR BUREAU OF INFORMATION.

**Rules for Correspondents.**

1. Every letter must be accompanied by the coupon to be cut from the back cover (inside page) of THE HOSPITAL, current issue, and must contain the name and address of the correspondent with pseudonym for publication if desired. Replies by post cannot be given save under exceptional circumstances at the Editor's discretion.
2. Letters from Approved Homes in reply to special needs published in the Bureau should state terms and full particulars, and be sent prepaid under cover to the Editor of the Bureau with name written across coupon for identification.
3. Proprietors of Homes which have not yet been entered on the List of Approved Homes, but have spare accommodation likely to suit special needs, are invited to write for an application form for registration. The fee for registration, which includes two announcements of the Home in the Bureau and other privileges, is 10s.
4. All communications to be addressed to the Editor of THE HOSPITAL, 28 Southampton Street, Strand, London, W.C. 2, and marked "Bureau of Information."

### INSTITUTION FACTS AND FIGURES.

### Social Work for V.A.D.s.

#### THE CHARITY ORGANISATION SOCIETY.

**By H. M. N.**

AMONG the societies which are on the alert to catch demobilised V.A.D.s on the rebound, so to speak, is the Charity Organisation Society, which offers a wide sphere of social work, both paid and voluntary. As there is probably no society which has suffered more criticism as to its ways of dealing with the poor, it is desirable to have some idea of its principles and methods before offering to work for it.

The C.O.S. is not a charitable Society in the usual meaning of the term; it is a Society for *organising* charity—a magnet which draws together existing lines of help so as to focus them upon the applicant. If, for instance, the applicant has been in service, former employers are approached, to see if they will unite in making up a small weekly pension; if he or she requires false teeth or a surgical instrument, co-operation is sought from the Samaritan Fund of any hospital attended, and also from the Hospital Sunday Fund, through the vicar or minister. It is only when other means of assistance have been exhausted that the C.O.S. sets about raising the balance by special appeals sent out to likely residents in the district.

In all but an infinitesimal number of cases the applicant must contribute something towards the cost; nor will the Society deal with any case in which near relations who are able to help refuse to do so. It must also be grasped at the outset that the C.O.S.'s decision as to whether a case is suitable for them to take up is based upon whether it is "helpable" and not whether it is "deserving"; whether the assistance which the Society would be able to obtain for the person concerned would do him permanent good, or whether, when it was exhausted, he would really be in the same plight as before. For instance, if a man has definite prospect of work at the end of a given period, the C.O.S. endeavours to tide over that period and to keep the home together. If the man is definitely out of work with no reasonable prospect of employment the Society will not attempt the support of the family for an uncertain period. Neither will it ever give "doles"; its motto is to help adequately or not at all. It follows therefore that many people who have referred applicants of excellent character to the C.O.S. are shocked and indignant because they are not helped. This does not imply a reflection upon the applicant, but indicates that he has failed in some particular to come within the scope of the Society's work.

It also follows that in order to decide which cases can be undertaken, and also to assess what payment can fairly be asked from the applicant himself, it is necessary to begin by making the fullest inquiries, so that the committee may have all possible relevant facts before them. These inquiries, embracing character, wages, the state of the home, and the earnings of all the members who contribute to its upkeep, etc., are another great cause of complaint against the C.O.S., and certainly they are extremely thorough. The C.O.S. claims that time and experience have confirmed its belief that they are also extremely necessary.

Enough has been said to show that the organisation of charity through the C.O.S. offers great scope for full-time workers and also for those who can only spare a few hours a week. There are appeals and letters of every description to be written, home visits to be paid, and applicants to be interviewed. As there are local offices in all London districts, and in many provincial towns, it is usually possible for voluntary helpers who wish it to work in their own neighbourhoods, though the really poor districts naturally look for help to the better-off ones. The telephone book will give London V.A.D.s the addresses of all London offices of the C.O.S., and no doubt the secretary of the one selected would arrange an interview. He or she would also be able to give particulars of training for the paid posts of the Society, or they could be obtained from Denison House, Vauxhall Bridge Road, London, S.W.

Previous articles appeared July 19 and Sept. 13.

### Hypodermic Syringes.

WHILE all glass syringes are cheap, we would not recommend them on the ground of economy. Only among the more expensive type of glass syringe can a watertight joint between nozzle and needle be guaranteed. When the barrel and nozzle are made in one piece, the syringe cannot be repaired if any part of it be broken; if, therefore, you prefer a glass syringe we suggest that you obtain one which is made in sections. For hospital use, the "Record" pattern syringe is difficult to beat. A 20 minim or 1 c.c. syringe costs about 9s. 6d.; although the initial outlay is greater, it is a reliable syringe, and the glass barrel, when broken, can be replaced for three or four shillings.—A. B. C.

### Question for October.

### REGULATING A PATIENT'S VISITORS.

Elaborate a plan whereby the number of patients' visitors may be regulated on visiting days, so that none may be standing about the corridors and all may see the patient in turn. Each patient to be allowed six visitors, but not more than two at a time.

**(A minimum payment of 10s. 6d. will be made for each published answer.)**

RULES.

The following rules must be observed:—

1. Contributions must be written on one side of the paper. Conciseness and terseness are desirable features. The MS. must bear the name and address of the sender and be accompanied by coupon to be cut from the back cover (inside page) of the current issue of The Hospital. A pseudonym must be chosen if the name is not to be published.

2. Contributions must be addressed to the Editor of The Hospital Institutional Worker Supplement, 28 & 29 Southampton Street, Strand, London, W.C. 2, should reach him before the end of the current month, and be marked in the left-hand corner "Facts and Figures."

### QUESTIONS INVITED.

In connection with our Question Box, we offer every month five shillings each for the best questions which are sent in for consideration. The questions may be on any institutional subject and concern any department, from the secretary's to the porter's, or the matron's to the domestic staff; the one essential is that they must be practical and deal with points that have a definite relation to hospital or institutional administration, upkeep, finance, or management. Points relating to artificers' work, housekeeping, the laundry, out-patients, and other practical matters will be welcome. It is hoped that thus, when difficult or doubtful points arise in the course of their work, institutional workers will be encouraged to put them in the form of a question and send them to the Editor, The Hospital Bureau of Information, 28 & 29 Southampton Street, Strand, London, W.C. 2, marked "Question Box."

The best questions will be published in due course and our readers will be given the opportunity of answering them. Thus all institutional workers may be able to co-operate with the view of helping the work and smoothing the difficulties of each other. In short, we want the Question Box of the *Institutional Worker* to be freely used by every worker habitually.

**The Editor will be glad to receive correspondence and to consider contributions upon all subjects relating to institutional work which affect the welfare of institutional workers.**

### ENQUIRIES AND ANSWERS.

### *THE SICK AND IN NEED.*

### War Service Gratuity.

We suggest that you apply, giving full details, to the Command Paymaster from whom you received your pay, or to the Matron-in-Chief of the Service under which you have served. We regret we cannot give postal replies.—K. S.

### Home for Poor Gentlewoman.

If the gentlewoman in whom you are interested is no longer able to work, we advise her to apply for admission to one of the following homes: The Belmont Home for Poor Gentlewomen, 14 Lewisham Park, S.E. Candidates must be over fifty and under sixty-five years of age, with an income of not less than £30 per annum and not more than £50. The inmates of the home pay 1s. per week and provide their board. The Incorporated Homes for Ladies, 29-31 Spencer Road, New Wandsworth, S.W. Applicants must be between fifty and seventy years of age and possess an income of not less than £26 and not more than £50 per annum. Each lady pays 1s. per month, furnishes and provides everything for her room. A donation of £25 is required on admission. In each case application should be made to the Honorary Secretary.—Mrs. H.

### Patient Suffering from Cancer.

There are a few beds provided for the use of patients, who may remain for life, at the Cancer Hospital, Fulham Road, Brompton, S.W., and we suggest that you write on behalf of the patient to the Secretary, who will, no doubt, advise her upon the matter.—K. S. C.

### *MISCELLANEOUS.*

### Books on Massage.

We think you will find "Theory and Practice of Massage," by Miss B. M. Goodall Copestake, most useful to you. The price now is 9s. and it is obtainable of The Scientific Press, Ltd., 28 & 29 Southampton Street, Strand, London, W.C. 2. For surface marking obtain "Landmarks and Surface Markings of the Human Body," by Rawlings, through the same publishers.—Masseuse.

### Nurses and Anæsthetics.

An excellent book dealing with the nurses' duties in relation to the administration of anæsthetics, by A. de Prenderville, has been recently published by William Heinemann. It may be obtained through The Scientific Press, Ltd., 28-29 Southampton Street, Strand, W.C. 2. The price is 3s. 6d.—Nurse.

### *EMPLOYMENT AND TRAINING.*

### Post required in a British Colony.

We suggest that you apply to the Overseas Nursing Association, Imperial Institute, London, S.W., for particulars. Its purpose is to provide trained nurses for private and hospital work in the British Colonies and Dependencies.—Penelope.

### Dispensing in Canada and Australia.

It is essential in all the provinces for dispensers to be registered after examination, but in some cases the diploma of any other competent examining body is accepted. We suggest that you write to the Secretary, Society of Apothecaries, Water Lane, Blackfriars, E.C., who will inform you which colonies accept their certificate.—Floral.

### Examination in the Nursing of Tuberculosis.

Lectures on tuberculosis are to be given at the Brompton Hospital, Brompton, S.W., during the months of October and November, the fee for which is £1 1s. Practical work can also be obtained; an examination is held at the end of the course and certificates granted. The fee, including the practical course, examination, and certificates, is £2 2s.—M. S. C. H.

### Temporary Post as Matron or Sister.

We are afraid we can do no more than advise you to answer the advertisements which appear each week in the nursing journals. Your position is an exceedingly difficult one, and we are sorry to say by no means singular just now. Your qualifications should, however, enable you to obtain a temporary post. Advertise your needs, stating your position.—D. B.

### Reduced Training for V.A.D.

There are a few hospitals in London who reduce their training of four years to three years for V.A.D.s, but none that we know of who will reduce the length of training to one year. Apply to some of the following institutions: University College Hospital, Gower Street, W.C.; St. George's Hospital, Hyde Park Corner, S.W., King's College Hospital, S.E., and St. Mary's Hospital, Paddington, W.—D. S.

### East London Nursing Society.

The address of the East London Nursing Society is Camperdown House, Half Moon Passage, Aldgate, E. It is affiliated to the Queen Victoria's Jubilee Institute for Nurses; the object of the Society is to nurse the sick poor of London in their own homes. Only fully trained nurses are employed; the salary offered is between £55 and £60 per annum, lodging and uniform.—Nurse B.

